# Phenotypic and genotypic resistance to antibiotics in *Staphylococcus aureus* strains isolated from cattle milk in Northern Kazakhstan

**DOI:** 10.14202/vetworld.2023.1815-1820

**Published:** 2023-09-14

**Authors:** Bakhit Muratovich Baymenov, Aitbay Kabykeshovich Bulashev, Gulzhagan Dzhambulovna Chuzhebayeva, Gulnur Kozyevna Aliyeva, Indira Saltanovna Beishova, Sabit Kabdyshevich Kokanov, Vitaly Anatolevich Raketsky

**Affiliations:** 1 Research Institute of Applied Biotechnology, A. Baitursynov Kostanay Regional University, Kostanai, Republic of Kazakhstan; 2 Department of Microbiology and Biotechnology, S. Seifullin Kazakh Agro Technical Research University, Astana, Republic of Kazakhstan; 3 Testing Center, West Kazakhstan Agrarian and Technical University Named after Zhangir Khan, Uralsk, Republic of Kazakhstan; 4 Department of Veterinary Medicine, A. Baitursynov Kostanay Regional University, Kostanai, Republic of Kazakhstan

**Keywords:** antibiotic resistance genes, cow milk, polymerase chain reaction, *Staphylococcus aureus* strains

## Abstract

**Background and Aim:**

*Staphylococcus aureus* is the most frequent and ubiquitous cause of mastitis in cows. In recent decades, antibiotic resistance has rapidly spread among infectious disease pathogens in Kazakhstan and globally. This study examined the phenotypic and genotypic resistance of *S*. *aureus* strains obtained from cattle milk to antibiotics.

**Materials and Methods:**

In 2021 and 2022, 675 cow milk samples were collected from 16 dairy farms in Northern Kazakhstan. *Staphylococcus aureus* was identified using culture and biochemical methods. The nature of antibiotic resistance was determined by the disk diffusion (DD) method. The distribution of antibiotic resistance genes was determined by polymerase chain reaction.

**Results:**

Among the obtained *S. aureus* isolates, high levels of resistance to β-lactam antibiotics (100%), tetracyclines (95.4%), fluoroquinolones (95.4%), and macrolides (60.92%) were observed. Meanwhile, the lowest levels of resistance were identified for sulfonamides (21.84%) and aminoglycosides (27.59%). All the obtained isolates were positive for the *nuc* gene encoding thermonuclease. The *blaZ*, *ermC*, and *tetK* genes were detected in 45.9%, 77%, and 83.9% of the studied *S. aureus* isolates, respectively.

**Conclusion:**

The results indicate a high prevalence of antibiotic resistance in *S. aureus* isolated from cows with clinical and subclinical forms of mastitis in Northern Kazakhstan. In addition, the prevalence of resistance was higher when evaluated by the DD method than when detecting the specific antibiotic resistance genes *blaZ*, *tetK*, and *ermC*, indicating the need for deeper analysis of the phenotypic and genetic determinants of antibiotic resistance.

## Introduction

*Staphylococcus aureus* is one of the main causative agents of contagious mastitis in cattle and its resistance to antibiotics is a global problem. *Staphylococcus aureus* carries several virulence factors and antibiotic resistance genes [1]. Antimicrobial therapy is important in controlling mastitis caused by *S. aureus*, but it has become less effective because of widespread antibiotic resistance [2]. *Staphylococcus aureus* strains exhibit adaptability to new conditions; therefore, monitoring the mechanisms of their virulence and antibiotic resistance is extremely important because this can facilitate the development of new treatment and prevention methods [3].

Resistance to antibacterial medications (ABMs) is of great socioeconomic importance and it represents a threat to national security. The uncontrolled use of antimicrobials, including the widespread use of antibiotics in veterinary medicine, animal husbandry, poultry farming, and the production and storage of livestock products, increases the risk of resistance at a global level [4–6].

Phenotypic methods make it possible to assess the presence of an enzyme related to ABM resistance, but they do not provide information about which of several hundred enzymes is present. Genetic methods cannot replace phenotypic methods in the routine testing of ABM susceptibility because new or previously unknown resistance mechanisms constantly arise and existing resistance genes are mobilized from ecological reservoirs and transferred through antimicrobial breeding [7]. However, genetic studies are important for analyzing the spread of genetic determinants of ABM resistance.

Prior studies [8–10] described examples of bacteria acquiring transient resistance to antibiotics without genetic changes. For example, bacteria live in an environment that does not provide sufficient nutrients for their metabolism, which is typical for bacteria in the growth inhibition phase. Another mechanism of ABM resistance found in some populations of microorganisms is persistence (natural insusceptibility to ABM) [8]. In addition, resistance to ABMs might be associated with the formation of biofilms that provide a protective barrier attributable to the polysaccharide matrix and presence of other cells, limiting the direct effect of antibiotics [9, 10].

One of the main sectors of the Kazakh economy is animal husbandry, in which dairy farming plays an important role [11]. In Kazakh farms, between 20% and 40% of cows are infected with mastitis [12]. Bovine mastitis can result in reduced productivity, loss of milk production, increased treatment costs, and mortality [13]. An analysis of studies in this area revealed the absence of a clear pattern in the distribution of genetic profiles of ABM resistance, which greatly complicates the prevention and treatment of mastitis.

The study aimed to determine the phenotypic and genotypic resistance to ABMs in *S*. *aureus* strains isolated from cattle milk in Northern Kazakhstan.

## Materials and Methods

### Ethical approval

Animal studies were conducted in compliance with biosafety and animal welfare standards. A positive conclusion from the Local Ethical Committee of the Research Institute of Applied Biotechnology of Kostanay Regional University named after A. Baitursynov, was obtained for conducting animal experiments (Protocol No. 1 dated May 19, 2020).

### Study period and location

The study was conducted from 2021 to 2022. Milk samples were collected from 16 dairy farms in the Northern region of Kazakhstan. Studies on the isolation and study of the phenotypic properties of *S. aureus* were carried out at the testing laboratory of the Research Institute of Applied Biotechnology of Kostanay Regional University named after A. Baitursynov, Kazakhstan.

### Bacteriological examination of milk samples

Milk samples (n = 675) were collected from cows with clinical and subclinical forms of mastitis. The selected milk samples were subjected to bacteriological examination and 87 *S. aureus* isolates were obtained and identified using molecular genetic methods. To identify subclinical forms of mastitis, the Draminski apparatus (DRAMIŃSKI S.A., Poland), which functions according to the electrical conductivity of milk, was used (http://www.draminski.com). To isolate and identify *S. aureus*, primary milk cultures were seeded in salt broth. The tubes with the cultures were incubated at 37°C for 24–48 h. After incubation, the culture was seeded on mannitol salt agar (HiMedia Laboratories, India). Typical colonies in the form of flat or convex yellow lemon-colored disks with smooth edges were seeded on the surface of meat–peptone agar (Biocompas-S, Russia). The hemolytic properties of the strains were determined on blood agar (HiMedia). To confirm the presence of coagulase-positive staphylococci, the rate of Gram staining and the ability of strains to coagulate rabbit blood plasma were measured, and their ability to ferment mannitol under anaerobic conditions was tested. Deoxyribonuclease activity was studied in DNase medium (HiMedia) with toluidine blue (HiMedia). Bacteria were confirmed to be *S. aureus* using the STAPHYtest 24 identification system (ERBA Lachema, Czech Republic) [14].

### Microbiological study

To isolate *S. aureus*, egg yolk high salt agar (Russia), Baird–Parker agar (Merck, Germany), and CHROMagar Mastitis (CHROMagar, France) were used. Biochemical identification of the cultures was performed using STAPHYtest systems (ERBA Lachema). The obtained isolates were identified by real-time polymerase chain reaction (RT-PCR) and sequencing of the 16S RNA gene.

### Testing of antibiotic susceptibility

Studies on antibiotic resistance were conducted using the disk diffusion (DD) method on Mueller–Hinton broth (MHA, Merck, Germany). The interpretation was performed following the recommendations of the European Committee on Antimicrobial Susceptibility Testing, version 9.0 [15] and methodological guidelines 4.2.1890–04 MU. Determination of the susceptibility of microorganisms to ABM was performed as described by Chief State Sanitary Doctor of the Russian Federation [16].

The disks were treated with the following antibiotics: Ampicillin (10 μg), amoxicillin (25 μg), benzylpenicillin (10 units), streptomycin (10 μg), cefoperazone (75 μg), cefoxitin (30 μg), kanamycin (30 μg), neomycin (30 μg), gentamicin (120 μg), tetracycline (30 μg), doxycycline (30 μg), ciprofloxacin (5 μg), norfloxacin (10 μg), erythromycin (15 μg), tylosin (15 μg), and sulfamethoxazole/trimethoprim (1.25/23.75).

### Identification of resistance genes

The genomic DNA of phenotypically identified *S. aureus* colonies was extracted by boiling [17] using PureLink Genomic DNA Kits (Thermo Fisher Scientific, USA) following the manufacturer’s instructions and stored at −20°C until further analysis. The target gene for identifying *S. aureus* by PCR was the thermonuclease (*nuc*) gene (77 bp), which was chosen because of its specificity [18–20]. The genotypic study of strains targeted genes related to resistance to β-lactam antibiotics (*blaZ*, 193 bp), macrolides (*ermC*, 142 bp), and tetracyclines (*tetK*, 167 bp). The primers for the target gene sequences were selected using the National Center for Biotechnology Information Primer-BLAST tool (https://www.ncbi.nlm.nih.gov/tools/primer-blast).

Polymerase chain reaction was performed in a 20-μL reaction mixture containing 3 mM magnesium and 0.2 mM of each nucleotide triphosphate. The concentrations of primers and probes in the reaction mixture were 200 and 400 nmol/L, respectively. The amplification protocol is presented in Table-1.

**Table-1:** Amplification execution protocol.

Block no.	Temp. (°C)	Time		Number of cycles	Optical measurement mode

		Min	S		
1	95	5	0	1	
2	94	0	10	40	
	60	0	20		FAM, JOE, ROX, TAMRA

As a positive control sample (PCS), pTG19-T plasmid vectors (Generay, Shanghai, China) containing target gene regions transformed into chemocompetent *Escherichia coli* DH5a cells were used. At this stage, the following processes were performed: Fragment amplification with PCR; electrophoresis on an agarose gel; purification of the PCR mixture according to the QI Aquick 28104 protocol (Qiagen, Valencia, USA); ligation of pTG19-T and the DNA fragment; transformation of the plasmid in *E. coli* into a DH5a strain; PCR screening of plasmids with primers; and preparation and isolation of plasmid structures. The production of PCS and synthesis of primers and fluorescence-labeled probes were performed at the National Center of Biotechnology Limited Liability Partnership (Kazakhstan, Astana, Z05K8A3).

### Statistical analysis

**Figure-1:**
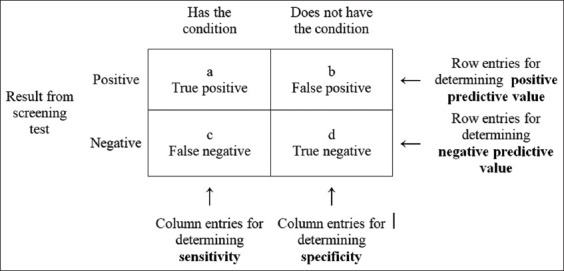
Accuracy of diagnostic tests.

To study the correspondence between the DD method and PCR, the diagnostic sensitivity (formula 1), specificity (formula 2), and the positive (formula 3) and negative (formula 4) predictive values (PPV and NPV, respectively) were calculated (Figure-1) [21].

1) Sensitivity = (a/[a + c]) × 100
2) Specificity = (d/[b + d]) × 100
3) Positive predictive value = (a/[a + b]) × 100
4) Negative predictive value = (d/[c + d]) × 100

The Kappa index was used to determine the degree of coincidence of the DD method with the detection of resistance genes by PCR [22]. The DD method was used as a reference for determining positive and negative results.

## Results

According to the results of antibiotic resistance tests, all 87 strains of *S. aureus* were resistant to at least two ABMs. In particular, all isolates were resistant to β-lactams, whereas 83 isolates (95.4%) were resistant to tetracyclines and fluoroquinolones. Meanwhile, 53 (60.92%), 24 (27.59%), and 19 isolates (21.84%) were resistant to macrolides, aminoglycosides, and sulfonamides, respectively (Table-2). Moreover, 3 (3.44%), 17 (19.54%), 46 (52.87%), 19 (21.83%), and 2 (2.29%) isolates were resistant simultaneously to two, three, four, five, and six ABM groups, respectively. In addition, 8 (9.19%), 9 (21.83%), 32 (36.7%), 19 (21.83%), 8 (9.19%), and 1 isolate (1.14%) was resistant to four, five, six, seven eight, and nine ABMs, respectively. In addition, 24 isolates exhibited phenotypic susceptibility to cefoxitin, whereas 13 isolates carried the *blaZ* gene and exhibited resistance to at least five ABMs.

**Table-2:** Antibioticogram of 87 *S. aureus* strains.

Species	Group ABM	Name of the ABM	Number of resistant strains	% of the total number of isolates	Number of resistant strains in the ABM group	% of the total number of isolates
*S. aureus* n = 87	β-lactams	Ampicillin	54	62.07	87	100
		Amoxicillin	48	55.17		
		Benzylpenicillin	46	52.87		
		Cefoperazone	48	55.17		
		Cefoxitin	24	27.59		
	Aminoglycosides	Streptomycin	11	12.64	24	27.59
		Kanamycin	4	4.60		
		Neomycin	7	8.05		
		Gentamicin	3	3.45		
	Tetracyclines	Tetracycline	51	58.62	83	95.4
		Doxycycline	50	57.47		
	Macrolides	Erythromycin	32	36.78	53	60.92
		Tylosin	28	32.18		
	Sulfonamides	Sulfamethoxazole/Trimethoprim	19	21.84	19	21.84
	Fluoroquinolones	Ciprofloxacin	51	58.62	83	95.4
		Norfloxacin	49	56.32		

*S. aureus*=*Staphylococcus aureus*, ABM=Antibacterial medications

### Polymerase chain reaction analysis of the *nuc, blaZ, ermC*, and *tetK* genes

After identifying the most conserved gene regions, primers and probes with similar physical characteristics were selected, permitting simultaneous amplification in a multiplex reaction (Table-3).

**Table-3:** Primers used in multiplex PCR.

Bacteria	Primer sequences (5′-3′)	Target genes	Gene ID	PCR product size (bp)
*S. aureus*	AATATGGACGTGGCTTAGCGT	*nuc*	DQ507382.1	77
	AGCCAAGCCTTGACGAACTAA			
	FAM-TGCTGATGGAAAAATGGTAAACGAAGC-BHQ1			
	AAGACGGTGTTCCAAAAGACT	*blaZ*	U58139.2	193
	ACACTCTTGGCGGTTTCACT			
	JOE-AGGTTGCTGATAAAAGTGGTCAAGCA-BHQ1			
	ATCGTGGAATACGGGTTTGCT	*ermC*	MF095627.1	142
	GTGAGCTATTCACTTTAGGTTTAGG			
	ROX-CGCTCATTGGCATTACTTTTAATGGCA-BHQ2			
	TCGATAGGAACAGCAGTATATGGA	*TetK*	HF679144.1	167
	GCAGATCCTACTCCTTGTACTAACC			
	TAMRA-TGAGCTGTCTTGGTTCATTGATTGCT-BHQ2			

PCR=Polymerase chain reaction, *S. aureus*=*Staphylococcus aureus*

Among the 87 phenotypically identified *S. aureus* isolates, the *nuc* gene was detected in all isolates using RT-PCR. The *blaZ* gene encoding resistance to β-lactams was found in 40 *S. aureus* isolates, of which 37 samples displayed resistance to benzylpenicillin as confirmed using the DD method. However, the *blaZ* gene was not detected in nine *S. aureus* isolates exhibiting resistance to benzylpenicillin. Three *blaZ*-positive *S. aureus* samples were susceptible to the indicated ABM.

Using the DD method as a reference, the diagnostic sensitivity, specificity, PPV, and NPV of PCR for the *blaZ* gene were 80.43%, 92.68%, 92.50%, and 80.85%, respectively, and the Kappa value was 0.73, indicating discrepancies in the classification of isolates as susceptible or resistant. Meanwhile, a statistically significant difference was detected between PCR and the DD method for detecting *ermC*. The *ermC* gene was detected in 67 *S. aureus* isolates, whereas resistance to macrolides was detected in 53 isolates (32 to erythromycin and 28 to tylosin) by the DD method. Phenotypic resistance to erythromycin was absent in 14 *ermC*-positive isolates, whereas the *ermC* gene was not detected in 12 tylosin-resistant isolates. The diagnostic sensitivity, specificity, PPV, and NPV of PCR in comparison with the DD method for detecting the *ermC* gene were 81.54%, 36.36%, 79.1%, and 40%, respectively, and the Kappa value was 0.18, indicating a significant discrepancy in the classification of isolates as susceptible or resistant.

Serious phenotypic resistance was observed to β-lactams and tetracyclines. Regarding the tetracycline-resistant isolates, 33, 32, and 18 were resistant to tetracycline, doxycycline, and both antibiotics, respectively. Meanwhile, the *tetK* gene was detected in 73 isolates exhibiting resistance to tetracyclines. The *tetK* gene was not detected in six *S. aureus* isolates displaying tetracycline resistance and four isolates exhibiting doxycycline resistance. The diagnostic sensitivity, specificity, PPV, and NPV of PCR in comparison with the DD method for the *tetK* gene were 87.95%, 100%, 100%, and 28.57%, respectively, and the Kappa value was 0.40, indicating a significant discrepancy between the methods in the classification of isolates as susceptible or resistant (Table-4).

**Table-4:** Results of the study of 87 *S. aureus* isolates in comparison of PCR with the DD method.

Genes	n	Sensitivity	Specificity	PPV	NPV	Kappa
*nuc*	87	100	100	100	100	1
*blaZ*	87	80.43	92.68	92.50	80.85	0.73
*ermC*	87	81.54	36.36	79.1	40	0.18
*TetK*	87	87.95	100	100	28.57	0.40

PCR=Polymerase chain reaction, *S. aureus*=*Staphylococcus aureus*, PPV=Positive predictive value, NPV=Negative predictive value, DD method=Disk diffusion

## Discussion

In recent decades, the resistance of infectious pathogens to ABMs has rapidly spread in Kazakhstan and worldwide. According to the results of this study, 100% of the *S. aureus* isolates were positive for the *nuc* gene encoding thermonuclease. The results revealed a correlation coefficient of 1 (perfect agreement) between the microbiological and PCR methods, indicating high specificity for detecting *S. aureus* using the *nuc* gene, consistent with the results of Alipour *et al*. [23].

The *S. aureus* isolates displayed high phenotypic resistance to β-lactam antibiotics. The *blaZ* gene was detected in 45.9% of the obtained isolates. The *blaZ* gene was not detected in nine *S. aureus* isolates with phenotypic resistance to benzylpenicillin, and three *blaZ*-positive isolates were susceptible to this drug. The reason for this discrepancy could be that resistant isolates lacking the *blaZ* gene have independent mechanisms unrelated to the acquired resistance gene, or it might be attributable to the existence of inadequate interpretation criteria when phenotypically susceptible strains possessed the *blaZ* gene and produced β-lactamase [24]. In addition, 24 isolates displayed phenotypic susceptibility to cefoxitin, whereas 13 carried the *blaZ* gene and exhibited resistance to at least five ABMs. According to prior research, the DD test for cefoxitin is applicable for detecting methicillin-resistant *S. aureus* (MRSA) with high sensitivity and specificity [25]. Most MRSAs synthesize β-lactamases, but they are also expressed by methicillin-susceptible *S. aureus* [26].

Phenotypic resistance to macrolides was detected in 60.9% of the isolates, whereas the *ermC* gene was detected in 77% of the isolates. All phenotypically erythromycin-resistant isolates were positive for the *ermC* gene. Fourteen *ermC*-positive isolates did not exhibit erythromycin resistance. The *ermC* gene was frequently detected, similar to previous findings by Russi *et al*. [27] and Mahfouz *et al*. [28]. The *ermC* gene was not detected in 12 tylosin-resistant isolates. It can be assumed that resistance to tylosin was caused by other antibiotic macrolide resistance genes not investigated in this study, such as *ermA* and *ermB* [29].

Phenotypic resistance to tetracyclines was detected in 95.4% of the isolates, including 33, 32, and 18 isolates that were resistant to tetracycline, doxycycline, and both antibiotics, respectively. These indicators of antibiotic resistance are consistent with the results of similar studies in this field [30]. The *tetK* gene was not present in six *S. aureus* isolates exhibiting tetracycline resistance and four isolates displaying resistance to doxycycline. In the remaining 73 tetracycline-resistant isolates, the *tetK* gene was detected. Resistance to tetracyclines might also be attributable to other determinants of resistance not investigated in this study, such as *tetL*, *tetM*, and *tetO*.

Thus, the rates of resistance in *S. aureus* isolates were generally higher for the DD method than for PCR-based detection of specific antibiotic resistance genes such as *blaZ*, *tetK*, and *ermC*. This result is most likely attributable to the limited study of genes for resistance to ABMs. In addition, it is assumed that one or more mechanisms of ABM resistance are present among *S. aureus* isolates. The presence of resistance genes, despite the absence of phenotypic resistance, could be explained by the presence of genetic determinants of antibiotic resistance, which is only one of multiple pathways of antibiotic resistance.

## Conclusion

Our study revealed that *S. aureus* isolates from cattle with mastitis in Northern Kazakhstan exhibited a high level of phenotypic resistance to β-lactams, tetracyclines, fluoroquinolones, and macrolides widely used in veterinary practice. They frequently carried genes associated with resistance to antibacterial drugs (*blaZ*, *ermC*, *tetK*). The results highlighted a high level of antibiotic use associated with the application of medicines in dairy farms in this region. In the analysis of antibiotic resistance of *S. aureus* strains, a statistically significant difference was observed in the rates of resistance between the DD method and PCR. This indicates that the PCR method should be cautiously used in clinical studies. This result can also be explained by the fact that only a few ABM resistance genes were investigated, and further research is required. Thus, monitoring ABM resistance profiles is important for studying trends regarding the phenotypic and genotypic factors of *S. aureus* resistance.

## Authors’ Contributions

AKB and GDC: Conceptualization, design, and planning of the study, data collection and analysis, critical review of the paper, and final approval. BMB, GKA, and ISB: Conducting research, statistical analysis, and first draft. SKK and VAR: Sampling and delivery of samples and conducting research. All authors have read, reviewed, and approved the final manuscript.
